# Age and preoperative pain are major confounders for sex differences in postoperative pain outcome: A prospective database analysis

**DOI:** 10.1371/journal.pone.0178659

**Published:** 2017-06-06

**Authors:** Hua Zheng, Alexander Schnabel, Maryam Yahiaoui-Doktor, Winfried Meissner, Hugo Van Aken, Peter Zahn, Esther Pogatzki-Zahn

**Affiliations:** 1 Department of Anesthesiology, Tongji Hospital, Tongji Medical College, Huazhong University of Science and Technology, Wuhan, China; 2 Department of Anesthesiology, Intensive Care and Pain Medicine, University Hospital Muenster, Muenster, Germany; 3 Department of Anesthesiology and Intensive Care, University Hospital Wuerzburg, Wuerzburg, Germany; 4 Institute for Medical Informatics, Statistics and Epidemiology, University of Leipzig, Leipzig, Germany; 5 Department of Anesthesiology and Intensive Care, Jena University Hospital, Jena, Germany; 6 Department of Anesthesiology and Intensive Care Medicine; Palliative Care Medicine and Pain Management, Berufsgenossenschaftliches Universitätsklinikum Bergmannsheil GmbH Bochum, Ruhr University Bochum, Bochum, Germany; Tokai Daigaku, JAPAN

## Abstract

**Objectives:**

Current literature is in disagreement regarding female sex as a risk factor for pain after surgery. We hypothesized, that sex differences exist but that they are influenced by certain factors. Here, we investigated the influence of sex for different clinically relevant postoperative pain (POP) outcome parameters and evaluated the role of assumed confounders for sex differences.

**Methods:**

From 1372 screened patients undergoing orthopedic surgery at the university hospital of Muenster between March 2010 and June 2011, 890 patients were included. The validated International Pain Outcomes questionnaire was used to assess the role of sex for several aspects of POP including pain severity, physical and emotional functional interference as well as the patient’s perceptions of the care they received on the first day after surgery. Assessed confounders were age, preoperative chronic pain, anesthetic technique employed and surgical procedure. All statistical analyses were performed with SPSS Statistics Software 22.

**Results:**

Linear regression analysis demonstrated that sex was a statistically significant risk factor for “worst pain since surgery”. Additionally, significant sex differences in “time spent in severe pain”, “feeling anxious due to pain”, “feeling helpless due to pain” and “opioid consumption since surgery” could be identified. An univariate general linear model showed that “age” and “preoperative pain” were significant confounders for sex differences. Further descriptive subgroup analysis revealed consistent sex differences for several POP outcome variables especially in patients older than 50 years or patients with preoperative chronic pain. However, sex differences disappeared in younger patients and in patients without preoperative pain.

**Discussion:**

Our data confirmed that sex differences exist in pain intensity and frequency, pain interference with feelings and opioid consumption during the first 24 hours postoperatively. However, sex differences were significantly influenced by the factors “age” and “preoperative pain”. These findings may in part explain why clinical studies get different results related to sex differences and renders specific awareness in older women and female patients with preoperative chronic pain.

## Introduction

More than 240 million surgical procedures are performed worldwide every year [1] and pain guidelines and evidence-based recommendations have been made to improve postoperative pain (POP) management [2]. However, postoperative pain is still insufficiently treated with more than 50% of patients suffering from moderate to severe pain early after surgery [3]. One of the reasons for this unsatisfactory situation might be an underestimated role of risk factors for a poor postoperative pain outcome. Although increasingly studied, clinical relevance of most risk factors are not determined yet.

One factor increasing the risk to experience more severe pain after surgery is the (female) sex of the patient. Sex specific differences in pain in general are well described in the literature [4–6]. However, the situation for POP is not yet clear. In some studies women tended to report more pain both at rest and during movement after surgery compared to men, but this trend is not consistent throughout the literature [7–9]. Even more controversial is the effect of sex on opioid consumption and opioid-induced side effects after surgery. Although morphine seems to be more effective in women compared to men [10], clinical trials investigating sex differences of perioperative morphine consumption showed conflicting results [7–9, 11].

A recently published large database analysis demonstrated that sex—apart from age and preoperative chronic pain—is a possible risk factor for severe POP with a mean differenced of 0.23 on a visual analogue scale (VAS) from 0–10 between males and females [12]. However, the clinical relevance of this finding needs further examination mainly due to the small total difference and reporting of only one clinical outcome parameter (“worst pain intensity”), but not other pain-related outcomes including pain frequency, interference with activities and feelings, opioid consumption and perceptions of care. Furthermore, other factors such as age, preoperative pain or psychological factors likely affect the severity of acute postoperative pain. If these factors are independent risk factors or interact in any way–e.g. with the risk factor sex–is basically not knon yet. Together, clinical data focusing on the exact role of the risk factor “sex” for poor pain outcome after surgery, on confounding factors influencing sex as a risk factor and on the clinical relevance of sex as a rsik factor are currently limited [13].

The major aim of the present study was to investigate sex differences in POP outcome (beyond worst pain after surgery), the influence of possible confounding factors on sex differences and the clinical relevance of these results in additional descriptive subgroup analyses.

## Materials and methods

The present study was conducted as part of the EU funded „Pain Out”project, an international multicenter benchmarking-project for the improvement of acute POP treatment (http://www.pain-out.eu) [14]; Institutional Review Board approval for the whole project was given in Jena, Germany (2723-12/09) and for the project site in Muenster (University Hospital of Muenster) by the local ethic committee Muenster, Germany (2009-936-b-S). Data presented here were collected prospectively between March 2010 and June 2011—exclusively in the University Hospital of Muenster, Germany, which is a specialist hospital for orthopedic surgery with several subspecialties providing supra-regional service for several orthopedic indications.

### Data collection and definition of outcome data

Patient outcome data were collected using the validated international pain outcome (IPO) questionnaire [15]. The IPO contains 13 questions which cover five areas of outcome measurement in POP–pain intensity, interference of pain with activities and feelings, analgesic side-effects and the patient’s impression of the care they received. The IPO questionnaire includes the following items: Least pain since surgery (numerical rating scale (NRS) 0–10); Worst pain since surgery (NRS 0–10); Percentage of time in severe pain (0–100%); Pain interference with activities in bed (NRS 0–10); Pain interference with activities out of bed (NRS 0–10); Pain interference with breathing/coughing (NRS 0–10); Pain interference with sleep (NRS 0–10); Emotional impairment due to pain: anxious (NRS 0–10); Emotional impairment due to pain: helpless (NRS 0–10); Adverse effects: nausea (NRS 0–10); Adverse effects: drowsiness (NRS 0–10); Adverse effects: itching (NRS 0–10); Adverse effects: dizziness (NRS 0–10); Percentage of pain relief from all treatments combined (0–100%); Participation in decision making (NRS 0–10); Satisfaction with pain treatment (NRS 0–10); Presence of a persistent painful condition for 3 months or more before the surgery (yes/no) including Intensity of the persistent pain before surgery (NRS 0–10).

A research assistant screened patients on the orthopedic wards at the university clinic of Muenster one day after surgery and informed patients in written form as well as orally to get verbal agreements. Due to the observational nature of the study and its characteristic as a quality improvement project, written informed consent was–at the time the study was performed—not required (ethic committees of the University of Jena and University of Münster). As defined by the Pain-Out Project, patients were included, if they were inpatients (≥ 18 years), consented to participate in the project and able to fill out the questionnaire by themselves. Exclusion criteria were refusal to participate, undergone repeat surgery, cognitive impairments or language deficiencies. After inclusion, patients filled out the IPO and the research assistant collected demographic data (sex, age, weight, height, nationality, place of birth, language of participant), medical history data (e.g. preoperative chronic pain) and process data (e.g. type of operation according to ICD-9, anesthesia techniques, analgesia technique, analgesic consumption etc.) from the patients’ charts in standardized fashion according to the Pain Out protocol. All data were assessed on the first day after surgery. The collected data were anonymized and then entered into the Pain Out Registry using a web-based tool as described recently [16].

The primary outcome variable we used here to assess sex differences and confounders for sex differences was the intensity of worst pain since surgery (numeric rating scale (NRS) 0 = “no pain”– 10 = “worst pain possible”). As secondary outcome we investigated the time the patient spent in severe pain since surgery (NRS 0% = “never in severe pain”– 100% = “always in severe pain”), functional disability due to pain (NRS 0 = “did not interfere”– 10 = “completely interfered”), anxiety and helplessness caused by the pain (NRS 0 = “not at all”– 10 = “extremely”), cumulative opioid consumption since surgery calculated by conversion of opioid requirements into morphine equivalents (ME), the degree of pain relief through pain treatment (NRS: 0% = “no relief”– 100% = “complete relief”), wish for more pain treatment (“yes or no”) and satisfaction with the results of pain treatment (NRS 0 = “extremely dissatisfied”– 10 = “extremely satisfied”).

### Perioperative pain management

All patients were treated according to the German postoperative pain guidelines and the standard procedures in the University Hospital of Muenster. In general, patients without contraindication received a premedication with a benzodiazepine (e.g. midazolam) and a non-opioid analgesic (e.g. etoricoxib, paracetamol or metamizol). Patients with preoperative chronic pain continued taking their daily pain medication perioperatively. According to our standard operating procedure and in agreement with the orthopedic surgeons most surgery was carried out under general anesthesia (GA) alone or combined with a regional anesthetic (RA)—as patient controlled analgesia (PCA) via a catheter (e.g. epidural) or single shot (e.g. interscalene nerve block). In a small number of cases patients underwent surgery with a RA (e.g. spinal anesthetic) alone. All patients receiving GA were treated intraoperatively with sufentanil or remifentanil and with prophylactic antiemetics (dexamethasone 4mg and/or ondansetron 4mg) according to the postoperative nausea and vomiting (PONV) score. Pain scores at rest and movement (NRS 0–10) were assessed in the postanesthesia care unit (PACU) and on the ward. Most patients on the ward received a non-opioid at fixed time intervals. Pain triggers for rescue analgesia were NRS at rest > 3 or NRS at movement > 5. Patients without RA received an opioid on demand (e.g. immediate–release hydromorphone). After medium-sized and major procedures (e.g. open joint surgeries and large bone surgeries), a controlled released opioid was added. All patients receiving RA received continuous infusion of a local anesthetic (ropivacaine 0.2%) alone (e.g. interscalene nerve catheter) or a local anesthetic in combination with an opioid (e.g. lumbar epidural analgesia with ropivacaine 0.2% plus sufentanil 0.75 μg/ml according to our current standard operating procedure for regional anesthetic procedures (Table 1). Postoperative iv-PCA was used rarely and only if planned regional analgesia techniques failed or pain management with oral opioids was insufficient.

**Table 1:** Details of postoperative management including a continuous regional anesthetic technique.

Analgesic Technique	Drugs applied	Basal infusion rate	Demand Dose	Lockout-Interval
**Patient-controlled epidural analgesia**	Ropivacaine 0.2% or bupivacaine 0.175% + Sufentanil 0.175 μg/ml (for patients <70 yr and without chronic opioid medication)	5 ml/h	2 ml	20 min
**Interscalene/axillary nerve catheter**	Bupivacaine 0.175%	Weight based manner (Half of max. recommended dose (0.4mg/kg KG) as basal infusion rate, other half as two possible boli within one hour)		30 min
**Femoral/sciatic nerve catheter**	Ropivacaine 0.2%	Weight based manner (Half of max. recommended dose (0.5mg/kg KG) as basal infusion rate, other half as two possible boli within one hour)		30 min

### Statistical analysis

In order to get homogeneous groups we re-evaluated the ICD 9 surgical codes within the data set and classified all surgical procedures into three groups (bone surgery, joint surgery and soft tissue surgery).

In a first step we carried out a linear regression analysis in order to determine the influence of possible predictors (“sex”, “age”, “preoperative chronic pain”, “anesthetic technique” (GA alone, RA alone, GA combined with RA) and “surgical procedure (bone surgery, joint surgery, soft tissue surgery)”) on worst POP since surgery.

In a second step we investigated the interaction of the above mentioned predictors with sex differences of worst pain intensity using a univariate general linear model (ANOVA).

Finally, in order to determine the clinical relevance of these results, we calculated means for all outcome parameters in all patients and then separately in women and men, respectively. Dichotomous data (e.g. number of patients requesting more pain treatment) were reported as absolute number (and %) and significance was calculated with the χ^2^ test. Demographic data (e.g. age) and outcome data rated on a NRS scale (most other data) were calculated as mean (± standard deviation (SD)). Sex related differences were compared with the nonparametric test for two unrelated samples (Mann-Whitney U Test). Statistical significance was determined using an α of 0.05. All statistical analyses were performed with SPSS Statistics 22 (IBM, Chicago, IL).

## Results

**Fig 1:**
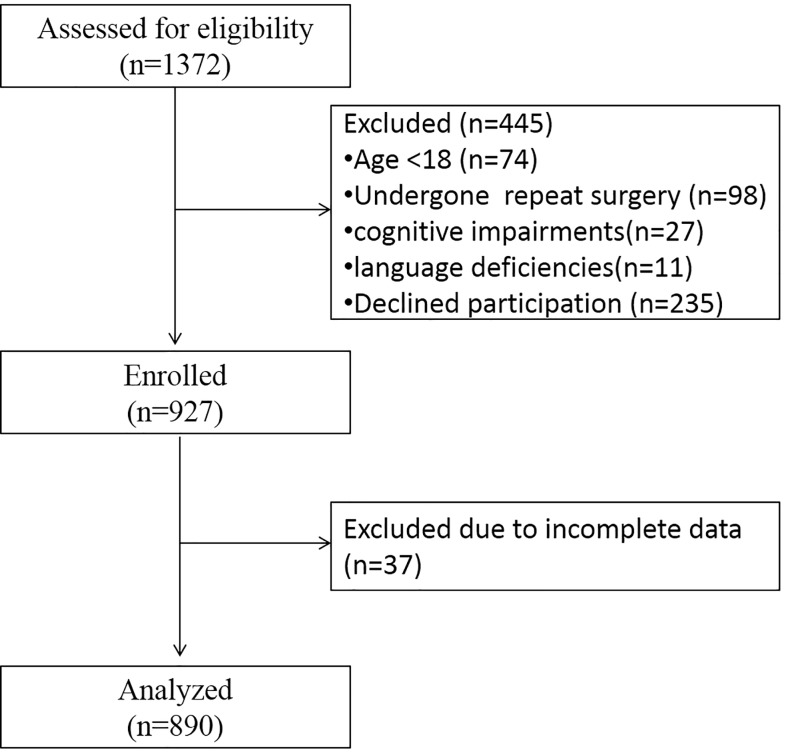
Patient flow during enrollment and analysis.

From 1372 screened inpatients undergoing orthopedic surgery in the university hospital of Muenster between March 2010 and June 2011 a total number of 890 (n_women_ = 403; n_men_ = 487) were included (Fig 1). The demographic data of the excluded patients were not different from the included patients.

### Demographic data

Demographic data of included patients are summarized in Table 2. The mean age of participants was 48.5 (± 17.0) years; women (50.9 ± 16.7 years) were slightly older than men (46.5 ± 17.0 years). Preoperative chronic pain was more frequently reported by women. With the exception of soft tissue surgery the sex distribution of the applied surgical procedures was comparable. In general most patients received a GA without RA (610 patients), 180 patients received a combination of GA and RA and 86 patients received RA alone. From 14 patients there were no data available regarding the applied anesthetic technique.

**Table 2:** Demographic data (as n (%)).

	Women (n = 403)	Men (n = 487)	p Value
**Age (years)**			
**<50 years**	176(39.6%)	269(60.4%)	<0.001
**≥50 years**	226(50.9%)	218(49.1%)	
**Preoperative chronic pain**			
**Patients with preoperative chronic pain**	275 (52.0%)	254 (48.0%)	<0.001
**Patients without preoperative chronic pain**	122(34.8%)	229(65.2%)	
**Surgical procedure**			
Bone surgery	154 (46.7%)	176 (53.3%)	0.214
Joint surgery	189 (46.6%)	217 (53.4%)	
Soft tissue surgery	59 (38.8%)	93 (61.2%)	
**Anesthetic technique**			
General anesthesia	274 (44.9%)	336 (55.1%)	0.248
Regional anesthesia	34 (39.5%)	52 (60.5%)	
General and regional anesthesia	90 (50.0%)	90 (50.0%)	

Sex related differences were compared with χ^2^ Test.

### Predictors for worst postoperative pain and identification of confounding variables for sex differences

Firstly, we performed a linear regression analyses in order to identify major predictors for worst pain intensity since surgery. As a result, age, existence of preoperative chronic pain, sex and anesthetic technique were significant predictors for worst POP since surgery (Table 3).

**Table 3:** Results of the linear regression analysis investigating possible predictors for worst postoperative pain since surgery.

Predictor Variable	p—Value	β	Lower bound	Upper bound
**Age**^**¥**^	**< 0.001**	-0.176	-0.714	-0.319
**Existence of preoperative chronic pain**	**< 0.001**	0.157	0.493	1.249
**Sexª**	**0.005**	-0.095	-0.888	-0.158
Anesthetic technique^*^	**0.030**	-0.072	-0.568	-0.029
**Surgical procedure**	0.261	Could not be included in model		

¥ grouped in 20 year intervals, in increasing order

ª Reference category is “male”

* GA and GA + RA compared to RA only

Secondly, we tried to identify confounding variables for sex differences in POP presumably influencing the predictive strength of sex. By using a univariate general linear model we demonstrated that the factor “age” and “preoperative chronic pain” significantly influenced sex differences of the primary outcome "worst POP since surgery" (Table 4). The other investigated factors (anesthetic technique and surgical procedure) were not identified as being significant confounders (Table 4).

**Table 4:** Results of the ANOVA investigating the interaction of the factors “age”, “preoperative pain”, “anesthetic technique” and “surgical procedure” with sex differences of the outcome “worst postoperative pain since surgery”.

Explanatory Factor	P value	
	Females	Males
**Age** *	0.282	**<0.001**
**Existence of chronic preoperative pain**	**0.034**	**0.006**
**Anesthetic technique**^**#**^		
**GA**	0.532	0.087
**GA with RA**	0.557	0.964
**Surgical procedure**^**Δ**^		
**Bone surgery**	0.684	0.076
**Joint surgery**	0.420	0.053

*up to 50 years old compared with 50^+^

^#^compared with RA only

^Δ^compared with soft-tissue surgery

### Sex differences in primary and secondary outcome data and influence of confounding variables

According to the results above, we investigated the sex differences of primary and secondary POP outcomes and the influence of age and preoperative chronic pain in descriptive subgroup analyses (Table 5).

**Table 5:** Results of sex differences in postoperative pain outcome and subgroup analysis focusing on the influence of age and preoperative pain.

Outcome	Sex	All Groups	Age Groups		Preoperative Pain Groups	
			<50 years	≥50 years	with	without
**Worst pain since surgery**	Women	5.75 ± 2.61*	5.85 ± 2.59	5.68 ± 2.85*	5.97 ± 2.72*	5.24 ± 2.71
	Men	5.20 ± 2.70	5.58 ± 2.61	4.72 ± 2.73	5.50 ± 2.60	4.89 ± 2.78
**Time spent in severe pain**	Women	34% ± 25%*	34% ± 24%*	34% ± 26%*	37% ± 26%*	27% ± 23%
	Men	27% ± 23%	27% ± 23%	26% ± 22%	29% ± 23%	24% ± 22%
**Interference of pain with in-bed activities**	Women	4.65 ± 2.83	4.68 ± 2.83	4.64 ± 2.83*	4.97 ± 2.71	3.92 ± 2.98
	Men	4.38 ± 2.83	4.81 ± 2.82	3.85 ± 2.76	4.75 ± 2.73	3.98 ± 2.91
**Feeling anxious due to pain**	Women	2.66 ± 2.53*	2.69 ± 2.49	2.65 ± 2.57*	2.98 ± 2.60	1.90 ± 2.17
	Men	2.18 ± 2.39	2.30 ± 2.44	2.04 ± 2.33	2.58 ± 2.46	1.73 ± 2.22
**Feeling helpless due to pain**	Women	3.13 ± 3.05*	3.01 ± 2.95*	3.24 ± 3.14*	3.59 ± 3.20*	2.12 ± 2.44
	Men	2.31 ± 2.66	2.37 ± 2.69	2.23 ± 2.63	2.74 ± 2.78	1.81 ± 2.43
**Cumulative opioid consumption(mg/kg)**	Women	0.39 ± 0.50*	0.39 ± 0.48	0.39 ± 0.51*	0.45 ± 0.55*	0.25 ± 0.34
	Men	0.31 ± 0.41	0.36 ± 0.44	0.24 ± 0.37	0.32 ± 0.42	0.30 ± 0.41
**Relief from pain**	Women	62% ± 26%	66% ± 24%*	59% ± 27%*	59% ± 26%	69% ± 26%
	Men	62% ± 28%	60% ± 28%	64% ± 27%	59% ± 27%	64% ± 28%
**Wish for more treatment was “Yes”**	Women	19% (72/388)	22% (37/169)	16% (35/218)	22% (58/267)	11% (13/117)
	Men	17% (80/471)	19% (48/260)	15% (32/211)	20% (49/244)	14% (31/225)
**Satisfaction with pain treatment**	Women	7.62 ± 2.75	7.62 ± 2.60	7.60 ± 2.88	7.49 ± 2.77	7.96 ± 2.69
	Men	7.53 ± 2.79	7.47 ± 2.61	7.61 ± 3.01	7.34 ± 2.93	7.74 ± 2.62

Results are presented as mean (± standard deviation) or relative numbers (% (n/N)). Groups were compared using Mann-Whitney U Test or χ^2^ Test (“wish for more treatment”).

* P<0.05 women vs. men

#### Sex differences in primary postoperative pain outcome

The mean pain score for “worst pain intensity since surgery” of the whole population was 5.45 (±2.73), whereas women reported significant higher worst pain intensity scores compared to men (p = 0.002) (Table 5). The subgroup analysis showed that older women (≥ 50 years) reported higher pain scores for “worst pain intensity since surgery” than older men (p<0.001). In contrast there were no significant differences within younger men and women (< 50 years) (Table 5). The subgroup analysis focusing on the influence of preoperative pain demonstrated that women had higher pain scores compared with men in the group of patients with preoperative chronic pain (p = 0.023), but not in the group of patients without preoperative chronic pain (Table 5). In addition, patients with preoperative chronic pain experienced higher postoperative pain intensity than patients without preoperative chronic pain (5.74 ± 2.67 vs. 5.01 ± 2.75; p<0.001).

### Sex differences in secondary postoperative pain outcome

#### A. Time in severe pain

The mean amount of time spent in severe pain was 30% (±24%) where women reported a longer amount of time in severe pain compared to men (p<0.001) (Table 5). The subgroup analysis focusing on the influence of age demonstrated that in both age groups women were significantly longer in severe pain (<50 years: p = 0.006; **≥**50 years: p = 0.001) (Table 5). The subgroup analysis according to preoperative pain showed that only in the group with preoperative pain women spent a significant longer time in severe time compared to men (p = 0.001). In contrast there was no sex related difference between women and men in the group without preoperative pain (Table 5). Moreover, patients with preoperative chronic pain experienced higher postoperative pain frequency compared to patients without preoperative chronic pain (33% ± 25% vs. 25% ± 22%; p<0.001).

#### B. Functional disability and mood during postoperative pain treatment

In all patients postoperative pain interfered with physical activities in bed like turning, sitting etc. with 4.50 (±2.83); women and men were comparably affected (p = 0.168) (Table 5). The anxiety score due to pain was 2.40 (±2.46) and the helplessness score due to pain was 2.68 (±2.87) in all patients. Women reported significantly higher anxiety (p = 0.003) and helplessness scores (p<0.001) due to pain compared to men (Table 5). The subgroup analysis investigating the influence of age demonstrated that only older women reported a significant higher impairment score of pain interference with physical activities in bed compared to men (p = 0.003). Furthermore this group of women showed significant higher anxiety (p = 0.012) and helplessness scores (p = 0.001) compared to men, whereas younger women reported only significantly higher helplessness scores (p = 0.018) (Table 5). Subgroup analysis focusing on the influence of preoperative pain showed that only women with preoperative pain reported significant higher “helplessness” scores compared to men (p = 0.004) (Table 5).

#### C. Cumulative opioid consumption

The cumulative postoperative opioid consumption (ME) in all patients was 0.346 mg/kg (± 0.456); women received significantly more ME compared to men (p = 0.009) (Table 5). The subgroup analysis comparing sex differences in different age groups demonstrated that only in the older age group this difference (women received a higher amount of opioids compared to men) was significant (p<0.001). In contrast, there was no sex related difference between younger women and men regarding to postoperative morphine consumption (Table 5). Women with preoperative pain required significantly more opioids (ME) compared to men (p = 0.006); however ME consumption in female and male patients without preoperative pain was comparable (Table 5).

#### D. Pain relief received

The mean amount of pain relief received was 62% (±27%); women received a similar amount of pain relief compared to men (P = 0.944) (Table 5). The subgroup analysis according to age showed that in the older age group (≥ 50 years) women received a significantly lower amount of pain relief compared to men (p = 0.040). In contrast in the younger age group women experienced a higher amount of pain relief than men (p = 0.025) (Table 5). Pain relief scores showed comparable results between both sexes in the subgroup analysis according to preoperative pain (Table 5).

#### E. Patients’ satisfaction with postoperative pain treatment

17.7% of all patients would like to have received more pain therapy; this was similar between women and men (p = 0.590) (Table 5). The mean satisfaction with the pain management in all patients was 7.57 (±2.77); women and men were both comparably satisfied with the pain management (p = 0.670) (Table 5). Both subgroup analyses (age, preoperative chronic pain) showed no significant differences of wish for more treatment and satisfaction (Table 5).

## Discussion

In this large sample of patients after orthopedic surgery we show that female sex is a significant predictor for worst pain since surgery. Besides pain intensity, we also identified significant sex differences in pain frequency, pain interference with feelings and opioid consumption during the first 24 hours postoperatively. Furthermore, for the first time, we demonstrated that preoperative chronic pain and age are major confounders of sex related differences in worst POP since surgery and determined certain special subgroups (especially older women and women with preoperative chronic pain) as being more or less susceptible to experience high pain scores after surgery.

### Sex differences in postoperative pain outcome

Several studies investigated sex as a possible predictor for worst POP intensity; however, results are sometimes not that strong and some studies even do not show sex differences [17–20]. An older qualitative systematic review summarizing the available literature concluded that sex seems not to be a consistent predictor for POP and analgesic consumption “as traditionally believed” [21]. However, more recently published data investigating procedure-specific risk factors for the development of severe POP strongly support a role of sex—apart from preoperative chronic pain and age—as one major predictor [12]. Another recent large retrospective study confirmed this by showing a higher risk of females to report more severe pain events; however sex differences were somehow small with odds ratios between 1.14 and 1.16 [22]. Here, we demonstrate that sex is as well a predictor for worst POP since surgery similar to those earlier clinical trials showing an effect on POP [7–9, 12, 22]. New are our additional findings on clinical aspects related to pain, for example that women are significantly longer in severe pain after surgery. We suggest that this factor might be even more clinically important; a longer time in severe pain might compromise early mobilization and physical therapy. In addition, a longer time in severe pain during the early postoperative period contributes to the development of chronic postsurgical pain [23, 24] and might explain in part, why females are at higher risk to develop chronic POP compared to males [25].

Furthermore, in our study women received 25.8% more opioids compared to men during the first 24 hours after surgery. This result is consistent with some other clinical trials investigating sex differences in opioid consumption in the PACU [7, 8]. However, two meta-analyses investigating sex differences of opioid analgesia in patients receiving a patient controlled intravenous opioid analgesia for POP treatment reported a higher analgesic efficacy of morphine in women compared to men [10]. The latter difference might be explained by the fact that women might show an increased benefit from self-control of POP treatment, which is supported by higher anxiety and helplessness scores in woman compared to men in our study. Together, the results obtained here confirm earlier results from large real-life observations that older patients report lower postoperative pain [12] with adding the new finding of decreased need for opioids in older men only.

Nevertheless, the observed sex differences in POP outcome were only small in our study. Furthermore, other analyzed pain related outcome variables (pain relief received, whish for more treatment and overall satisfaction with pain management) did not show any significant sex difference. This might indicate that the sex differences for the pain related outcome parameter, for example pain intensity ratings, are not that clinical relevant for females. For instance, females seem to be not really “affected” by their higher pain ratings (because satisfaction ratings and whish for more treatments were similar in males and females) suggesting that sex is not that relevant for the general aspect of POP. Furthermore, this might indicate that pain intensity is not the best (and only) outcome parameter relevant for a qualified pain management.

### Confounder for sex differences in postoperative pain outcome

We demonstrate for the first time that age and preoperative chronic pain (but not “anesthetic technique” or “surgical procedure”) might be major confounders for sex differences. These findings possibly explain—at least in part—why sex alone has not been found consistently as a risk factor for POP in other studies [21].

The subgroup analysis focusing on the clinical relevance of this result revealed that the sex differences in pain intensity, impairment of pain interference with physical activities and anxiety scores were more pronounced in the older group of patients, in contrast to the younger age group where these results were not statistically significant. More specifically, older women received 62.5% more opioids compared to older men, but they reported an even lower amount of pain relief compared to men. This interesting finding might be explained by the fact that other factors, like psychological variables (anxiety and helplessness scores) and physical impairment, may have a greater influence on pain relief in older patients compared to the younger group. Recently it has been shown, that psychological variables, like anxiety, pain catastrophizing, and emotional illness representation are stronger predictors than age and other clinical variables for persistent postsurgical pain following hysterectomy [26]. Therefore, psychological support and specific interventions by the caregivers may help to improve pain management, especially in older women undergoing orthopedic surgery.

The results of another subgroup analysis showed that only in patients with preoperative chronic pain sex differences were significant. Women with preoperative chronic pain report significant higher worst pain scores since surgery, spend a significant longer time in severe pain, show higher helplessness scores and require significant higher amounts of opioids compared to men with preoperative pain. In addition, patients with preoperative chronic pain experienced higher postoperative pain intensity and frequency than patients without preoperative chronic pain. The latter finding might be the reason for sex differences in patients with preoperative chronic pain, because sex differences appear to be more pronounced when postoperative pain is poorly managed. This is supported by a recently published trial, which demonstrated that sex differences seem to be diminished in women and men treated with an epidural analgesia for major surgical procedures indicating that effective pain management is able to overcome sex related differences [27]. Women with preoperative pain might need more effective pain management than other patients. Future studies should investigate methods in order to improve the outcome in this group of patients.

### Limitations

The results of this analysis are based on patient data of one single university hospital with an own standard of POP treatment, which might influence the generalization of our results. On the other hand, the single center data may have the strength by comparing a group of uniform treatments (similar surgeons, similar postoperative care and analgesic management, etc.). Patients analyzed within this database mostly were treated at least 24 hours after surgery within the hospital. Therefore, our results might not be transferable to outpatient surgery. We did not analyze the influence of preoperative opioid intake on POP outcome. However, it is well known that opioid naïve patients might have a different sensitivity to opioids than opioid-dependent patients, which might have influenced the opioid consumption. This together with other factors related to preoperative pain needs further investigation in the future. Because the opioid consumption might be influenced by the sex of the prescribing physician [28, 29], results focusing on opioid consumption might be influenced by this bias. However, our data were collected during daily routine. Therefore, this possible bias could not be controlled for needs to be investigated in the future. Additionally, due to lacking data we can not exclude that there were sex or age related differences in patients who had contraindications for the normal perioperative pain management treatment described within the methods. This might have influenced the results of the subgroup analysis. Treatment related confounders, although limited due to data collection within one single hospital and by applying standardized pain management protocols cannot be excluded as well. Furthermore, the present data only reflect the first 24 hours following surgery. Therefore, the calculated outcomes might be influenced directly by surgery or anesthesia and our results might be overestimated. However, it is well known from the literature that the first postoperative day represents the day with the highest pain scores during postoperative care and might be therefore the most important one even for sex differences [27].

## Conclusion

The current prospective database analysis confirmed that female sex is a significant predictor for worst POP since surgery. Accordingly, significant sex differences were seen in other POP-related outcome parameters like time in severe pain, pain interference with feelings and opioid consumption. However, parameters like received pain relief or satisfaction were not significantly different in males and females indicating that higher pain scores in females do not result in disaffection and sex differences depend on the outcome parameter assessed. We further demonstrated that age and preoperative pain are predictors for worst POP since surgery as well as clinically relevant confounding factors for sex differences for worst POP. More specifically, subgroup analyses revealed that especially older women (≥50 years) and women with preoperative pain might be at higher risk for suffering from worse POP outcome. The latter findings might explain the conflicting results for sex being a predictor for POP in earlier studies. Apart from that, older women and women with preoperative pain probably require more clinical awareness during postoperative care by providing multimodal pain treatment including perioperative psychological support.
